# Development and Characterization of Novel Polyurethane Films Impregnated with Tolfenamic Acid for Therapeutic Applications

**DOI:** 10.1155/2013/178973

**Published:** 2013-09-01

**Authors:** Hilal Istanbullu, Sofia Ahmed, Muhammad Ali Sheraz, Ihtesham ur Rehman

**Affiliations:** Department of Materials Science and Engineering, The Kroto Research Institute, University of Sheffield, North Campus, Broad Lane, Sheffield S3 7HQ, UK

## Abstract

The present study deals with the preparation of polyurethane (PU) films impregnated with a nonsteroidal anti-inflammatory drug, tolfenamic acid (TA). Solvent evaporation technique has been employed for the preparation of TA-PU films in two different ratios of 1 : 2 and 1 : 5 in Tetrahydrofuran (THF) or THF-ethanol mixtures. The prepared films were characterized using X-Ray Diffraction (XRD), Differential Scanning Calorimetry (DSC), Fourier Transform Infrared Spectroscopy (FTIR), Scanning Electron Microscopy (SEM), and release studies. The results indicate transformation of crystalline TA to its amorphous form. The degree of crystallinity changes both by increasing the polymer concentration and solvent used for the film preparations. The release profiles of TA were also found to be affected, showing a decrease from approximately 50% to 25% from 1 : 2 to 1 : 5 ratios, respectively.

## 1. Introduction

Polymer films are generally used for therapeutic purposes and a number of workers have reported the use of different polymeric films for the delivery of various therapeutic agents via different routes [1–6]. Among the variety of polymers available, polyurethanes (PU) are a family of polymers that are widely used as biomaterials in clinical applications. Their use not only in the medical field but also in other commercial applications is significant in many areas, since their easy structure manipulation allows the control of their chemical, physical, and mechanical characteristics [7–10]. PU are used in a wide range of biomedical applications due to their excellent physical and mechanical properties and relatively good blood compatibility [8–12]. They also possess great diversity due to their different chemical compositions and properties such as elasticity, tolerance in the body, durability, and compliance, which are generally superior to other biomaterial polymers [9, 12–14]. Recently, it has been reported that PU films prepared through solvent evaporation technique were effective in the controlled in vitro delivery of curcumin against human lung cancer cells [4].

**Figure 1:**
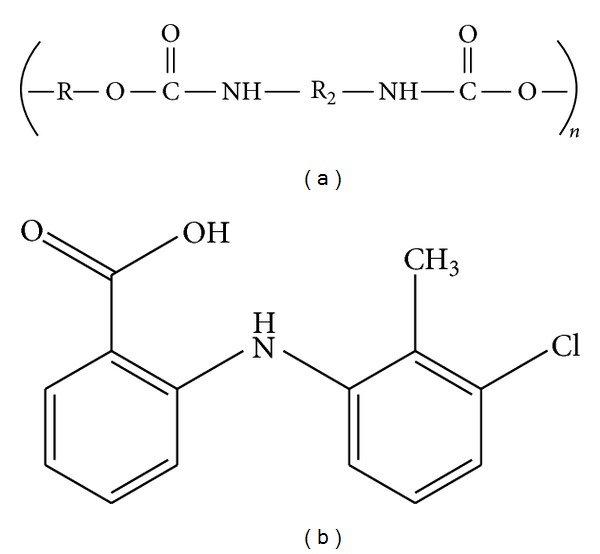
Structures of (a) PU and (b) TA.

Polyurethanes are among the many commercially important classes of polymers. The term “polyurethane” is more one of convenience than of accuracy, since these polymers are not derived by polymerising a monomeric urethane and are derived from the reaction of cyano (–NCO) and hydroxyl (–OH) groups to form urethane linkage. PU is a class of polymers, which contains a significant number of urethane groups contained within the molecule, although not necessarily repeating in a regular order [11]. A urethane group is formed by the chemical reaction between a hydroxyl and isocyanate group (Figure 1(a)).

PU contains intermolecular urethane linkages that are formed due to the condensation reaction of polyisocyanates and polyols, and most commonly diisocyanates and diols are used to obtain linear PU. Typically PU possesses three monomers: a diisocyanate, a macroglycol (diol), and a chain extender [7, 10, 13]. Polymeric materials may be dissolved either in pure solvents or in mixtures of different solvents. Mixtures are used in order to enhance the solvency power of the primary solvents. Tetrahydrofuran (THF) is found to be one of the best solvents for PU with a polymer interaction parameter value of 0.78 [15].

Tolfenamic acid (2-[(3-chloro-2-methylphenyl)amino]benzoic acid) (Figure 1(b)) is a nonsteroidal anti-inflammatory drug (NSAID) and is used in the treatment of migraine, dysmenorrhoea, rheumatoid arthritis, or osteoarthritis, and so forth [16, 17]. Recently, it has been discovered that this drug can slow down the progression of Alzheimer disease [18, 19]. In addition, it also possesses novel anticancer properties and a number of workers have employed tolfenamic acid (TA) successfully against variety of cancers [20–24]. TA occurs in crystalline form and is insoluble in water [17]. It has a bioavailability of only 60%–75% [25, 26]. One of the most common ways of increasing a drugs' solubility and bioavailability is converting it to an amorphous form.

A number of methods have been reported to make drugs amorphous and hence more soluble and bioavailable [27–33]. But often it has been observed that the metastable amorphous forms have the tendency of recrystallization during storage, which is the major drawback in their preparation. One of the widely used approaches for making the crystalline drug amorphous and avoid recrystallization is to complex it with a suitable polymer in optimum concentration. Polymers having the tendency to form hydrogen bonds with drugs are usually used for this purpose. Furthermore, the technique employed for complexation also affects the solid state properties of the crystalline drug particles [27–37].

The aim of this study has to evaluate the changes in the physical state properties of TA after its complexation with PU through solvent evaporation technique. As mentioned above both PU and TA have gained significant medical and pharmaceutical importance in the recent past. In view of their invaluable potential this is the first time when such combination has been reported. It is envisaged that the impregnation of TA in the polymeric matrix and the study of its release pattern may help in the development of novel therapeutic films for clinical applications.

## 2. Experimental

### 2.1. Materials

TA (99.97%) was purchased from Sigma-Aldrich Company Ltd. (Dorset, UK), PU resins (Z3A1, polyether-urethane) from Biomer Technology Ltd. (Runcorn, UK), THF (99.99%) and ethanol (99.99%) from Fisher Scientific Inc. (Loughborough, UK). All other chemicals and solvents used in this study were of analytical grade and purchased from Sigma-Aldrich Company Ltd. (Dorset, UK).

### 2.2. Preparation and Storage of Drug-Polymer Films

TA-PU films in 1 : 2 and 1 : 5 ratios were prepared through solvent evaporation technique by two different methods.

#### 2.2.1. Method I

In the first method, TA (1% w/v) and PU solutions (2% and 5% w/v) were prepared in THF. The drug solution was added in equal volumes in the polymer solutions in order to obtain the desired ratios, that is, 1 : 2 and 1 : 5. A measured volume was spread over the glass slide (76 × 26 mm) by the help of a glass pipette and kept for drying at room temperature in a fume cupboard (AFA 1000, Clean Air Limited, Bolton, UK) with an air flow rate of 1.50 m/sec.

#### 2.2.2. Method II

In the second method, TA solution (1% w/v) was prepared in ethanol and PU solution (2% and 5% w/v) in THF. Both solutions were mixed together in equal volumes in similar pattern to method I and poured over a glass slide using a glass pipette and dried as described above.

Pure polymer films (2% and 5%) were also prepared in the similar manner that served as control. The polymer solutions with more PU concentration, that is, 5% were found to be more viscous as compared to PU solutions of 2%. All the prepared films were protected from light throughout the experiments and stored in a desiccator containing silica gel at room temperature for up to 3 months.

### 2.3. Characterization Studies

As described above, the aims and objectives of this study have been to study the drug-polymer interaction and any changes in the solid state properties of TA. Therefore, the prepared films were thoroughly characterized by employing different analytical techniques to study these physical and chemical interactions.

### 2.4. X-Ray Diffraction (XRD) Analysis

The XRD patterns for the films were obtained with STOE STADI-P high-resolution diffractometer (Darmstadt, Germany) using Cu K-alpha1 radiation (wavelength = 1.54051 Å). Data was collected over the 2*θ* range of 5–60° for 60 min by using proprietary STOE WinX^POW^ software.

### 2.5. Differential Scanning Calorimetry (DSC)

The samples were studied for their thermal behaviour by DSC 6 (PerkinElmer, USA) using pin-hole aluminium pans with lids. The samples were weighed accurately (3 ± 0.1 mg) and heated from 30 to 250°C at a rate of 10°C per min. Nitrogen with a flow rate of 20 mL/min was used as a purge gas and indium and zinc standards were used for the calibration of the instrument. Pyris 7.0 software was used for data analysis.

### 2.6. Fourier Transform Infrared (FTIR) Spectroscopy

FTIR studies were performed using a Nicolet Nexus FTIR spectrophotometer (Thermo Fisher Scientific Inc., USA). The thin films were analysed in transmission mode, accumulating 64 scans at 4 cm^−1^ resolution in the spectral range of 4000–400 cm^−1^. The spectral data was analysed using the OMNIC 7.4 software.

### 2.7. Scanning Electron Microscopy (SEM)

SEM studies were performed by using Inspect F microscope (FEI, Holland) with an accelerating voltage of 5 KV. The samples were mounted on a 0.5 inch aluminium stub with the help of 12 mm double-sided adhesive carbon tabs (Agar Scientific, UK). The mounted samples were then outlined with silver solution and subjected to carbon coating using Speedivac carbon coating unit (Model 12E6/1598) to make them electrically conductive. The magnification of the microscope was selected as required for the detailed study of morphological changes of the samples.

### 2.8. Release Studies

The release studies of the TA-PU films were performed on a USP type II paddle dissolution apparatus (Model VDA-1D, Veego Instruments Corporation, India). Phosphate buffer (0.2 M, pH 7.2, 500 mL) was used as dissolution medium while temperature and stirring conditions were maintained at 37 ± 0.5°C and 50 rpm, respectively. All films were prepared in a manner to contain same amount of TA (i.e., 25 mg). Each time 5 mL of the sample was withdrawn from the vessel at suitable time intervals and an equal volume of the dissolution medium was added in order to maintain the sink condition. The concentration of TA in the withdrawn samples was determined by the UV spectrophotometric method according to the method described by Thybo et al. [31] using an appropriate blank of phosphate buffer with polymer.

## 3. Results and Discussion

### 3.1. Appearance of TA-PU Films

Equal volumes of the solutions were used in the preparation of all films. However, TA-PU films prepared by two different methods showed some differences in their appearance. The films prepared by method I (both drug and polymer in THF) were found to be more transparent with smooth surface whereas the films prepared by method II (drug in ethanol and polymer in THF) appeared opaque with slightly rough surface. No signs of recrystallization or changes in appearance took place in all the films during storage for up to 3 months in a desiccator.

### 3.2. Characterization Studies

#### 3.2.1. XRD Studies

In order to confirm the changes in the physical properties of the crystalline TA, the films were studied using XRD technique. XRD is usually considered as the most definite method of detecting and quantifying the crystalline/amorphous nature of the sample. XRD shows strong characteristic diffraction peaks for crystalline solids whereas diffused and hallow diffraction patterns for amorphous powders [30].

**Figure 2:**
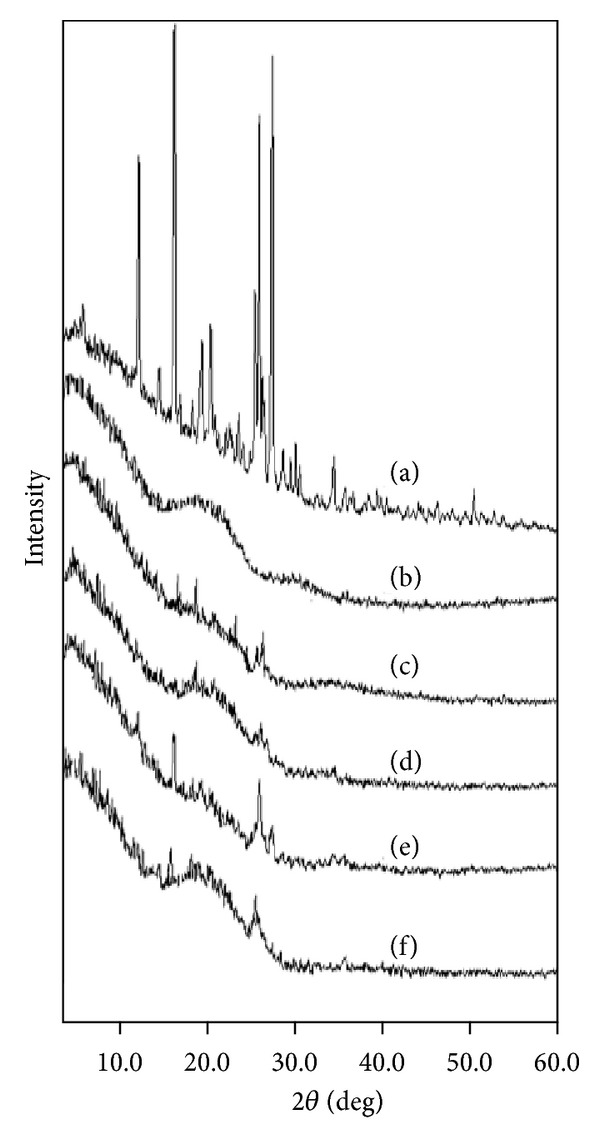
XRD patterns of (a) TA, (b) PU, TA-PU films prepared by method I in ratios (c) 1 : 2, (d) 1 : 5, TA-PU films prepared by method II in ratios (e) 1 : 2, (f) 1 : 5.

The pure crystalline TA shows characteristic diffraction peaks at 2*θ* values of 5.21°, 11.58°, 15.72°, 18.70°, 19.77°, 24.90°, 25.32°, 25.90°, and 26.81°, respectively (Figure 2(a)). In case of PU, a hallow diffraction pattern with no sharp peaks has been observed in the pure as well as in the control films thus indicating its amorphous nature. A typical diffraction pattern has been presented in Figure 2(b). The diffraction patterns of the 1 : 2 TA-PU films prepared by method I indicated a marked decrease in the peaks intensity of the crystalline TA and show very small peaks at around 15.72–18.70° and 24.90–26.81° whereas the remaining peaks of the drug have not been detected (Figure 2(c)). The diffraction pattern of 1 : 5 TA-PU films also shows similar type of result with comparatively less intense peaks in the same region (Figure 2(d)). This decrease in peak intensity clearly indicates that majority of drug has been transformed to the amorphous form.

The films prepared using ethanol for drug solubilisation (method II) showed similar type of diffraction patterns for 1 : 2 and 1 : 5 ratios, respectively, but the intensity of the peaks is comparatively higher to the films prepared in THF only (method I) (Figures 2(e) and 2(f)). Therefore, method I could be considered as more effective comparatively in converting crystalline TA to its amorphous form. Similarly, the difference in the diffraction pattern of the films indicates that the solvent also plays an important role in modifying the solid state properties of the drug in addition to the technique used and the nature of the polymer. THF evaporates more rapidly as compared to ethanol-THF mixture [38] and therefore the use of THF alone has resulted in greater amorphicity of the drug. The samples were further subjected to analysis by DSC, FTIR, and SEM techniques in order to confirm the results obtained by XRD.

#### 3.2.2. DSC Studies

**Figure 3:**
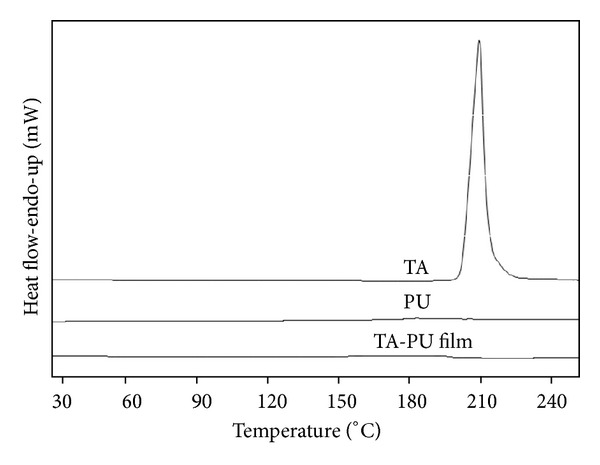
DSC thermogram of pure TA, PU, and their mixture. All TA-PU films showed similar type of pattern.

The DSC thermogram of pure TA and PU is given in Figure 3. The melting peak of pure TA has been observed at around 214°C whereas no peak has been detected in pure PU in the studied region of 30–250°C.

In the case of drug-polymer mixtures, no peak has been observed for any film in the region 30–250°C (Figure 3), which is not in agreement with the results of XRD. Increasing the sample amount up to 10 mg or cutting the films in very small fragments also found in vain. The possible reason for the absence of melting peak could be due to the impregnation or encapsulation of the drug within the viscous polymer matrix and PU with its high melting point has restricted the contact of the melting drug with the base of the aluminium pan thus leaving no signs of its melting to the detector. On the basis of these results, it has been concluded that DSC is not a useful technique in the current study in detecting the thermal behaviour of crystalline/amorphous form of TA in the polymer films.

#### 3.2.3. FTIR Spectroscopy

Spectroscopic methods due to their nondestructive nature can be used along with other solid-state techniques for quantitative analysis of pharmaceutical solids. FTIR spectroscopy is considered as a valuable technique to study the degree of crystallinity based on the measurement of characteristic peak intensity for its particular polymorphic crystal state [30]. Infrared technique is also very useful to study the hydrogen bond formation of polymers with different drugs [29, 31, 33]. Therefore, FTIR spectroscopy was used in this study to identify the possible interaction and complexation between TA and PU. It is envisaged that PU contain –COOH and –NH groups in its backbone, which may be involved in H-bonding with TA (Figure 1). As described above, PU is the polymer obtained from the polymerization of diisocyanate and polyol that contains electron donating sites; that is, C=O and –NH groups (Figure 1) both of which are sensitive to the formation of hydrogen bonds with the drug during complex formation [14, 39].

**Figure 4:**
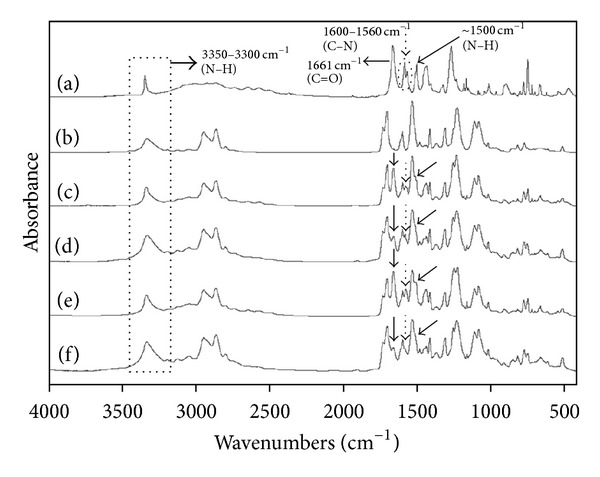
FTIR spectra of (a) TA, (b) PU, TA-PU films prepared by method I in ratios (c) 1 : 2, (d) 1 : 5, TA-PU films prepared by method II in ratios (e) 1 : 2, (f) 1 : 5. The dotted lines and arrows are highlighting the regions that show complexation and intermolecular hydrogen bonding between TA and PU films.

Peaks at 1661, 1590, 1575, 1500, 1270, and 749 cm^−1^ are the most intense, characteristic peaks of TA (Figure 4(a)). The FTIR spectrum of pure TA shows NH-stretching vibrations at around 3342–3340 cm^−1^. However, one of its other polymorphic forms gives similar stretching vibrations at 3324–3315 cm^−1^. Due to this reason, the spectral region between 3350 and 3300 cm^−1^ has been monitored for observing the spectral changes in the crystalline form of TA [31, 33, 40–42]. Similarly, PU is also known to exhibit its characteristic major NH peak at 3325 cm^−1^ (Figure 4(b)). Since pure TA and PU share the same spectral region to exhibit their characteristic peaks of NH-stretching vibrations, it was difficult to identify the changes taking place in the peak height of TA in the drug-polymer films at the spectral region of 3350–3300 cm^−1^. However, the films still show broadening of the peak in the same region that increases with increasing polymer concentration that is a clear indication of complexation between the drug and the polymer resulting in increased amorphicity of TA (Figures 4(c)–4(f)).

The other specific peaks for pure TA in the FTIR spectra could be observed at 1661 cm^−1^ for the stretching vibrations of C=O of carboxylic moiety, at 1600–1560 cm^−1^ for C–N stretching bands and at ~1500 cm^−1^ for NH-bending vibrations (Figure 4(a)). In the case of 1 : 2 (Figure 4(c)) and 1 : 5 (Figure 4(d)) TA-PU films, prepared by method I, a marked change has been noted in the peak intensity of the drug at 1661 cm^−1^, which is decreased with increasing polymer concentration suggesting that 1 : 5 TA-PU is more effective in converting the crystalline nature of the drug to its amorphous form. The C–N stretching bands have also been found to decrease in size with increasing polymer concentration particularly at ~1575 cm^−1^ in both 1 : 2 and 1 : 5 TA-PU films (Figures 4(c) and 4(d)). The NH-bending vibrations at around 1500 cm^−1^ of the drug could not be clearly observed in any of the TA-PU films due to the fact that the same bending vibrations are exhibited by PU in this region. However, still the appearance of small shoulder in the same peak in 1 : 2 ratios (Figures 4(c) and 4(e)) can be observed which eventually disappeared in 1 : 5 ratios (Figures 4(d) and 4(f)). The sharp peak at 749 cm^−1^ (Figure 4(a)) corresponds to the C–N deformation [41, 42] which has also reduced in the films (Figures 4(c)–4(f)) thus further confirming modification in the solid state properties of TA due to strong complexation and intermolecular hydrogen bonding between TA and PU.

The films prepared using ethanol for drug solubilisation (method II) have been found to demonstrate the similar spectral pattern as in case of TA-PU films in THF but with slightly stronger peak intensity as compared to the pure polymer suggesting the presence of more crystalline drug (Figures 4(e) and 4(f)). The FTIR data thus further supports the results of XRD.

#### 3.2.4. SEM Studies

**Figure 5:**
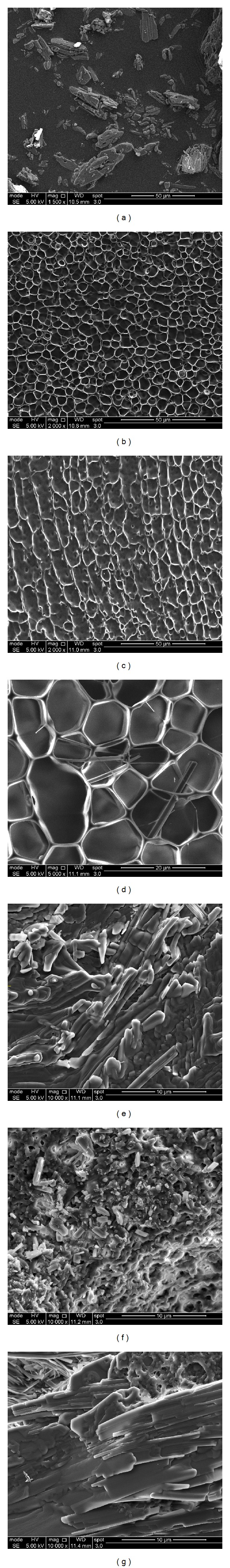
SEM images of (a) pure TA, (b) 2% PU, (c) 5% PU, TA-PU films prepared by method I in ratios (d) 1 : 2, (e) 1 : 5, TA-PU films prepared by method II in ratios (f) 1 : 2, (g) 1 : 5.

In order to observe any morphological changes in the drug-polymer particles, the films were analysed by SEM. The images of pure TA reveal particles of irregular size with rough surface (Figure 5(a)). The images of pure PU film show a honeycomb like structure which becomes more compact and chain like with increasing polymer concentration (Figures 5(b) and 5(c)). In case of films prepared by method I, the particles of TA on the surface of the film in 1 : 2 ratio can clearly be observed (Figure 5(d)). On the contrary, fused drug particles with altered physical appearance could be seen in film with 1 : 5 TA-PU ratio suggesting a better complexation between the TA and PU (Figure 5(e)). The 1 : 2 TA-PU film prepared by method II shows a mixture of irregular, smooth as well as rough surface particles indicating the partial conversion of crystalline TA into its amorphous form (Figure 5(f)). Similarly, the film with 1 : 5 TA-PU ratio also exhibits identical appearance of particles that are fused together but with slightly smoother surface (Figure 5(g)). These images further confirm the results of XRD and FTIR indicating the modification in the crystalline nature of TA. These images also show difference in surface morphology of the films when prepared by two different methods. More drug particles could be clearly observed in films prepared by method II as compared to method I films.

#### 3.2.5. Release Studies

**Figure 6:**
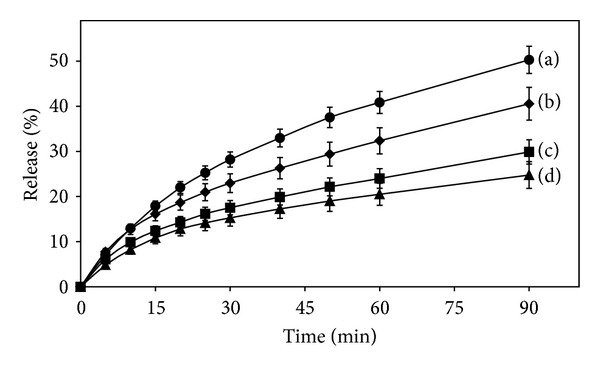
Release profiles of TA from TA-PU films. (a) 1 : 2 (method II), (b) 1 : 2 (method I), (c) 1 : 5 (method II), and (d) 1 : 5 (method I). The error bars represent the standard deviation where *n* = 3.

The comparative release pattern of TA from the different TA-PU films is presented in Figure 6. Almost 50% of the drug was released from the 1 : 2 ratio films in the initial 90 minutes prepared by method II. On the same time about 40% of the drug was released from the films of same ratio prepared by method I and about 30% and 25% from the films of 1 : 5 ratio prepared by methods II and I, respectively.

Films prepared by either method show that with increasing PU concentration the release of TA has been slowed down despite the fact that increased polymer concentration has converted more amount of the crystalline drug into its amorphous form. This could be due to the formation of viscous PU solutions, which entrapped or encapsulated the drug more effectively within its matrix and thus slowed down its release. On the other hand, the effect of method of preparation on drug release can also be observed as films prepared by method II showed better release of TA from the polymer matrix as compared to the films prepared by method I. This could be attributed to the presence of more TA particles on the surface of film that are evident from the SEM images (Figure 5).

## 4. Conclusion

Characterization of the films confirms the transformation of a significant amount of crystalline TA to its amorphous form, which is related to the methods of preparation and concentration of polymer. All the characterization data except DSC confirmed the drug-polymer interaction and it was observed that 1 : 5 TA-PU films prepared in THF (method I) were more efficient in converting the drug to the amorphous form as compared to the other ratio. The films prepared by dissolving TA in ethanol (method II) were found to contain comparatively more crystalline TA particles than the films prepared in THF only. This shows that the solvent used for drug solubilisation and its evaporation rate also play an important role in such type of solid-state transformations. Although films prepared by method I indicated greater degree of amorphicity but comparatively better release rates for tolfenamic acid (~50%) has been observed in films prepared by method II mainly due to the increased amount of drug present on the surface of the film. It has also been observed that TA release slowed down with increasing PU concentration due to the strong entrapment or encapsulation of the drug within the polymer matrix.

The present work thus provides useful guidelines to the researchers and formulators in developing such type of films that could be effectively used for therapeutic purposes in various clinical conditions.
